# Risk management of emergency service vehicle crashes in the United States fire service: process, outputs, and recommendations

**DOI:** 10.1186/s12889-017-4894-3

**Published:** 2017-11-17

**Authors:** David P. Bui, Keshia P. Porter, Stephanie Griffin, Dustin D. French, Alesia M. Jung, Stephen Crothers, Jefferey L. Burgess

**Affiliations:** 1 Mel and Enid Zuckerman College of Public Health, The University of Arizona, Drachman Hall, 1295 N Martin Ave, Campus PO Box: 245210, Tucson, AZ 85724 USA; 2 Johns Hopkins Bloomberg School of Public Health, Johns Hopkins Center for Injury Research and Policy, Baltimore, MD USA; 3 Center for Healthcare Studies, Department of Ophthalmology, Northwestern University Feinberg School of Medicine, Chicago, IL USA; 4 Department of Veterans Affairs, Center of Innovation for Complex Chronic Healthcare, Edward Hines, Jr. VA Hospital, Hines, IL USA; 5 Seattle Fire Department, Seattle, WA USA

**Keywords:** Risk management, Fire service, Safety and health, Traffic accidents, Injury prevention, Crash prevention

## Abstract

**Background:**

Emergency service vehicle crashes (ESVCs) are a leading cause of death in the United States fire service. Risk management (RM) is a proactive process for identifying occupational risks and reducing hazards and unwanted events through an iterative process of scoping hazards, risk assessment, and implementing controls. We describe the process, outputs, and lessons learned from the application of a proactive RM process to reduce ESVCs in US fire departments.

**Methods:**

Three fire departments representative of urban, suburban, and rural geographies, participated in a facilitated RM process delivered through focus groups and stakeholder discussion. Crash reports from department databases were reviewed to characterize the context, circumstances, hazards and risks of ESVCs. Identified risks were ranked using a risk matrix that considered risk likelihood and severity. Department-specific control measures were selected based on group consensus. Interviews, and focus groups were used to assess acceptability and utility of the RM process and perceived facilitators and barriers of implementation.

**Results:**

Three to six RM meetings were conducted at each fire department. There were 7.4 crashes per 100 personnel in the urban department and 10.5 per 100 personnel in the suburban department; the rural department experienced zero crashes. All departments identified emergency response, backing, on scene struck by, driver distraction, vehicle/road visibility, and driver training as high or medium concerns. Additional high priority risks varied by department; the urban department prioritized turning and rear ending crashes; the suburban firefighters prioritized inclement weather/road environment and low visibility related crashes; and the rural volunteer fire department prioritized exiting station, vehicle failure, and inclement weather/road environment related incidents. Selected controls included new policies and standard operating procedures to reduce emergency response, cameras to enhance driver visibility while backing, and increased training frequency and enhanced training. The RM process was generally acceptable to department participants and considered useful. All departments reported that the focused and systematic analysis of crashes was particularly helpful. Implementation of controls was a commonly cited challenge.

**Conclusions:**

Proactive RM of ESVCs in three US fire departments was positively received and supported the establishment of interventions tailored to each department’s needs and priorities.

**Electronic supplementary material:**

The online version of this article (doi: 10.1186/s12889-017-4894-3) contains supplementary material, which is available to authorized users.

## Background

Firefighting remains a dangerous occupation, resulting in over 63,000 injuries and an average of 81 deaths per year over the last 10 years [1–3]. Emergency service vehicle crashes (ESVCs) contribute to a significant portion of firefighter morbidity and mortality [4–6]. In 2014, there were over 14,000 ESVCs reported to the National Fire Protection Association (NFPA), resulting in approximately 600 injuries to firefighters [3]. In 2015, there were 13 vehicle-related fatalities including 8 ESVCs and 5 roadside struck by incidents, making ESVCs the 2nd leading cause of fatal injury in the United States (US) fire service for that year [2]. Furthermore, emergency service vehicles, with their large sizes and high speed operations, pose a significant hazard to civilians and public roadway motorists [5]. The cost of repair, property damage, injury and litigation as a result of ESVCs can result in considerable financial costs that are passed on to municipalities and the public [7, 8].

**Fig. 1:**
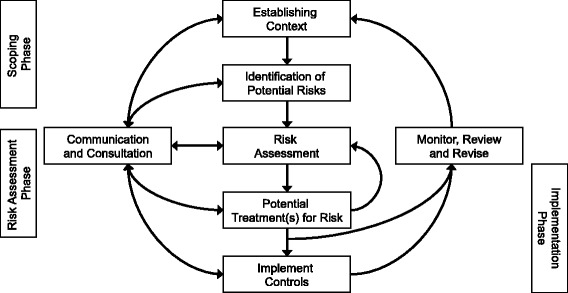
An overview of the Risk Management process, adapted from ISO 31000:2009 [11]

Risk management (RM) is a formalized proactive process that has been used in a range of occupational settings, including firefighting and mining, to reduce workplace hazards and injuries [9–11]. Organizations use formal RM to manage risks and hazards through identification, assessment, and prioritization of risks for mitigation and intervention [11]. RM follows a cyclical set of three phases, including 1) hazard scoping, 2) risk assessment, and 3) implementation of controls within a feedback loop (Fig. 1). While commonly required in other countries to protect the health and safety of industrial workers, formal RM is not as widely practiced in the US [12, 13], despite growing evidence suggesting it may effectively reduce a range of occupational hazards and injuries [13–15]. Widespread implementation of RM in Australia was associated with up to 78% reductions in lost-time injury rates in the Australian coal mining industry, compared to only a 20% reduction in the US coal mining industry which relied on compliance-based safety programs during the same timeframe [12]. An international comparison of fire service injury rates found that fire departments in the UK, with RM-based occupational safety regulations, had significantly lower fire service injury rates than US fire departments. RM has been previously used to reduce injuries and their economic consequences in the fire service. In the US, RM has been applied in the fire service to address injuries stemming from patient transport, fireground activities, and physical exercise [14]. Implementation of a risk management program aimed at improving the structure and management of physical exercise among recruit firefighters was associated with a statistically significant reduction in injury frequency and worker’s compensation claims cost [15]. These prior studies have shown that RM is was perceived to be useful to and valued by firefighters [13].

The goal of this study was to apply RM processes in three distinct US fire department settings to identify risks and hazards associated with ESVCs, determine potentially effective control measures, and evaluate the acceptability and utility of RM as a tool for mitigating ESVCs in the US fire service.

## Methods

Three fire departments (A, B, and C), representing a range of urban-rural geographic classifications, population served sizes, and career/volunteer firefighter mix, participated in this study. Department A originally requested the research to assist in reducing ESVCs. Departments B and C were already fire service partners with the researchers at the time of study initiation. Department A was a career fire department, serving a large major metropolitan area. Department B was a combination career/volunteer fire department serving a mostly suburban population. Department C was the only volunteer department in the study, serving largely rural and wildland areas. Department C is staffed by <100 volunteer firefighters, and serve a population of less than 100,000. The department covers approximately 100 mile^2^ that is 97% farmland, forestlands and recreational areas.

Teams within each department participated in a facilitated RM process delivered through focus groups and Delphi method panels. Three to six 2-h meetings were planned for each department to cover the scoping, risk assessment, and control implementation phases within the RM framework. Meetings were conducted in a combination of in-person and web-conference forums. All study procedures were approved by the University of Arizona Institutional Review Board.

### Scoping phase

The goal of the scoping process in meetings 1–2 was to clearly define and describe ESVCs, and the context and environments in which they occur. During these meetings, available crash data from participating departments were summarized with descriptive statistics and reviewed in facilitated discussions with the RM teams. Data collection and recordkeeping varied substantially by department, but generally included data on crash types, vehicle types, environmental conditions (e.g., time of day, weather, road type), response types (e.g., emergency or non-emergency response), driver maneuvers, driver rank, and a brief narrative.

RM teams from each department used these summary data to create a working list of hazards and risk factors for ESVCs. During the scoping meetings, the RM teams discussed the frequency of each crash type (e.g., side swipe, backing), crash conditions (e.g., during emergency response, bad weather), road hazards (e.g., deer crossing, environment), and driver actions (e.g., driver error, exiting stations) to create a list of high frequency risks to consider during the risk assessment phase.

### Risk assessment phase

**Fig. 2:**
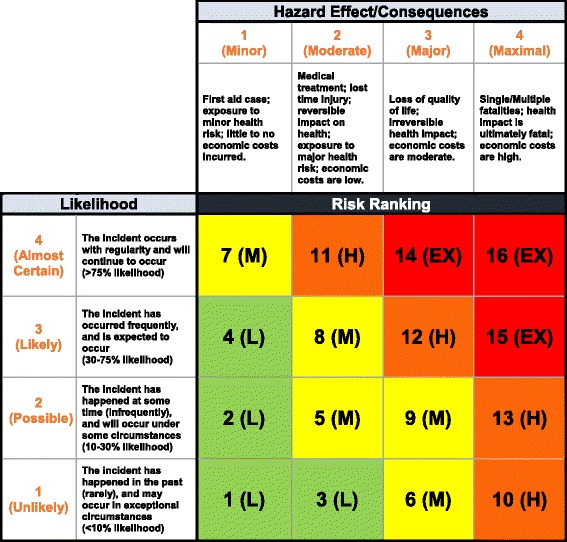
Hazard matrix used in ranking and prioritizing risks and hazards identified in the risk management scoping and discovery process

Meetings 3–4 were dedicated to formal risk assessment where teams carefully reviewed the risks and hazards defined during scoping and prioritized each for intervention. The risk and hazards were ranked using a risk matrix approach commonly employed in formal risk assessments [9, 10]. Risk matrices rank hazards based on a product of two domains: 1) the likelihood of the considered hazard to occur; and 2) the severity of bodily injury or harm produced should the hazard were to occur [10]. Likelihood was qualitatively and semi-quantitatively assessed using summary data reviewed during scoping. An ordinal likelihood score was assigned for each hazard on a scale from 1 (unlikely) to 4 (almost certain). Severity was qualitatively assessed based on consensus and scored on an ordinal scale from 1 (minor) to 4 (maximal). Given the high costs associated with emergency service vehicle collisions [7], RM teams were instructed to consider the burden of economic consequences in their assessment of incident severity (Fig. 2). Risks with a ranking of 1–4 were assigned as low priority, 5–9 as medium priority, 10–13 as high priority and 14–16 as extremely high priority (Fig. 2). Risks and hazards ranked as high to extremely high were prioritized for intervention.

### Implementation phase

The implementation phase was initiated in meetings 5–6. RM teams reviewed the high priority risks and hazards identified during the risk assessment phase and discussed possible interventions and controls (e.g., installing backup cameras, changing response policies) to address each risk and hazard. The study team provided a menu of common strategies and controls used in other departments to spur discussion of potential approaches and treatments. Risks were documented in a ‘risk register,’ which is a working document where each risk is clearly defined and ranked, and appropriate interventions and controls are designated and described for each risk. Teams decided which interventions to adopt and developed a schedule for implementation.

### Risk management process evaluation

Interviews and focus groups were conducted with 12 fire personnel across the three departments that were involved with the risk management process and meetings. Using a semi-structured interview guide (See Additional file 1), information was collected on the acceptability and perceived utility of the RM process in each department, as well as what were the perceived facilitators and barriers of successful implementation. Data were collected from two focus groups conducted with a total of nine individuals, and two interviews with three members of department leadership (one interview included two people) that occurred either in person or via telephone (for one department, leadership participated in the focus group rather than in an individual interview). Notes were captured from each interview and focus groups, which were reviewed by two members of the study team and coded based on themes that addressed our study aims. This paper focuses on analysis and evaluation of the RM process through the selection and implementation of controls; evaluation of control effectiveness is ongoing and will be reported in a subsequent manuscript.

## Results

### Departments and risk management teams

Crash data were only available for Departments A and B. Department C reported experiencing no crashes in recent years and did not have crash data for review during the scoping process. Instead of relying on crash statistics at Department C, we reviewed potential scenarios and incidents that could occur. The crash rates per 100,000 population served varied from 0 in Department C to a high of 13.9 in Department A (See Table 1).

**Table 1:** Profiles of fire departments participating in study, 2015

	Department A	Department B	Department C
Primary Geography	Urban	Suburban	Rural
Department Type	City, Career	County, Combination	District, Volunteer
Population Served, thousands	>1000	100–1000	<100
Area Size Served, sq. mi.	150–500	150–500	<150
Stations, *n*	>50	10–50	<10
Personnel Size, *n*	>1000	100–1000	<100
Fleet Size, *n*	>100	50–100	<50
Emergency Calls, *n*	>100,000	1000–100,000	<1000
Crash rates per			
100 personnel	7.37	10.45	0
10,000 emergency calls	5.99	16.76	0
100,000 population served	13.93	13.28	0
Risk Management Team, *n* (%)	23 (100)	8 (100)	5 (100)
Chiefs/Captains/Officers	7 (30)	2 (25)	1 (20)
Firefighters/Drivers	13 (57)	3 (38)	2 (40)
Administrators, Fleet	3 (13)	3 (38)	2 (40)
Risk Management Team Description	Head of safety chief, crash review panel consisting of firefighters/drivers, union representatives, general counsel, fleet manager	Head of safety chief, volunteer firefighters, risk management department representatives.	Department chief, fleet manager, volunteer firefighters, station administrator.

Each department recruited a broad range of personnel to participate in the RM process, including Battalion Chiefs, captains, frontline drivers, fleet managers, and administrators. Department A predominantly involved firefighters and front-line drivers, as well as union representatives. Although Department B invited volunteer firefighters to participate, only career firefighters and members from their liability risk management division participated in the process. Department C included volunteer firefighters in the RM meetings

### Identified risks and hazards

During the scoping phase, Department A reviewed data from 2418 crash records (occurring between 2008 and 2013) and Department B reviewed data from 197 crash records (2009 through 2013). Department C did not have any crash records to review and risks and hazards were determined through group discussion and consensus.

A range of risks and hazards were identified by each department and prioritized differently (Table 2). Responding with active lights and sirens (emergency response), on scene struck by incidents, and vehicle backing were generally regarded as a high-level risks/hazards for each department. Collisions occurring while exiting stations were deemed high priorities at Department C, but not at Departments A and B. Department A found certain maneuvers such as turning at intersections and sideswipes to be high priority incidents.

**Table 2:** Summary of risks, hazards and incident types and their priority ranking

Risks, Hazards and Incident Types	Department A	Department B	Department C
	Urban, Career	Suburban, Combination	Rural, Volunteer
On Scene / Struck By	**High**	Medium	**High**
Emergency Response (Lights & Siren)	**High**	**High**	**High**
Backing Up	**High**	**High**	Medium
Exiting Station	–	Medium	**High**
Insufficient Training / Education	**High**	**High**	**High**
Firefighter Driver Error /Distraction	**High**	Medium	Medium
Crossing/Clearing Intersections	Medium	Medium	–
Animal Related Incidents	–	Medium	**High**
Turning Maneuver Crashes	**High**	Low	–
Rear Ending	**High**	–	–
Sideswipe Incidents	Medium	–	–
Vehicle Failure (e.g. tire blowout)	Medium	Low	**High**
Low Visibility Incidents	Medium	**High**	**High**
Inclement Weather/Road Environment	Low	**High**	**High**

– solid lines indicate priority was not ranked; bolded text to indicate high ranked priorities. Rankings (high, medium, low) based on risk ratings provided by risk management teams at each department. Risk ranking is based on severity of a risk/hazard, the likelihood of it to occur and the economic impact of the event. Only risks, hazards and incidents shared by at least two departments are shown

Department A was concerned with rear ending civilian vehicles as well as being rear ended by other vehicles. Department C also described collisions with other responding fire service and law enforcement vehicles was a potential concern. In all departments, speeding and following too closely were noted concerns. Visibility related incidents were a medium to high priority concern for all departments and included concerns about seeing other vehicles and objects from the apparatus while driving and backing, as well as being seen by other vehicles while working on scene. In both Departments B and C, animal-related incidents, inclement weather and road environment were high priorities. Regarding drive behavior, errors and distractions were considered medium-to-high for all departments. All departments noted a high priority concern about insufficient training and continuing education for emergency vehicle drivers.

### Selected interventions

The interventions chosen by each department to be implemented varied and depended on the prioritized risks, costs, and feasibility. As such, a suite of interventions was selected that encompassed policy, training, education, engineering, and technological approaches.

#### Policy

Having identified emergency response to be an important risk factor for collisions, all departments chose to institute new department-wide standard operating procedures (SOPs) and policies aimed to reduce the use of unnecessary code 3 responses (i.e., lights and sirens). Since the risk of ESVCs is elevated during code 3 response [4, 16, 17], dispatch protocols were re-evaluated to identify non-emergency responses with low risk to loss of life and/or property which could be responded to without the use of lights and sirens. Department A released a new SOP requiring drivers to respond to non-emergency events (e.g., non-injury accident investigations, well-being checks, lock outs, etc.) without the use of lights and sirens. Department C also implemented a modified code 3 response policy to reduce the use of lights and sirens in their department. Department B elected to modify response policies to reduce emergency driving, but as of publication did not yet release the policy change to the department. To address backing related collisions, Department A planned to redesign existing SOPs for apparatus spotting and backing to emphasize the use of spotters. Department A also elected to enact a policy requiring removal of garage door remote control ‘clickers’ from stations to prevent incidents where garage doors were prematurely closed on apparatuses while exiting station garages.

#### Training and education interventions

Drivers’ training and train-the-trainer seminars were implemented at Department A. Departments A and B both chose to increase the use of driver simulator training systems they had previously acquired, however both departments report trainee nausea during simulation exercises to be common. To increase training frequency, Department A revised policies for remedial driver’s training for drivers involved in multiple vehicle incidents. They lowered the incident threshold from five non-serious incidents to three non-serious incidents per year before the offending driver is required to attend a 40-h fire service vehicle operator (FSVO) course, but the safety department retained discretion to prescribe training earlier if necessary. Notably, Department A did not have a dedicated area for driver training and decided to make acquisition of a training field/space a high priority.

Department B elected to implement the most training and education efforts, focusing on: proactive targeting of problem areas based on crash data, foul weather training, safe operation of station doors training, and pre-operation vehicle inspection training. Department B also emphasized deer collision avoidance education by implementing annual reviews of deer collisions and communicating avoidance strategies to the entire department through quarterly safety bulletins. The safety division analyzed and mapped previous deer incidents to identify hours and geographic hotspots where drivers should exercise caution while driving.

Department C did not elect to implement any training or education-based interventions.

#### Engineering interventions

Departments A and C both installed backup cameras on a small subset of vehicles to increase rearward visibility during backing maneuvers, particularly in ambulance units since spotting is not possible when patient care is being performed. Department B elected to install backup cameras and proximity sensors to address backing incidents. Department B also chose to purchase in-cab headsets to facilitate in-cab verbal communications as well as driver-to-spotter communications during backing maneuvers.

#### Vehicle data recorders

As part of larger project goals, all departments agreed to explore the use of vehicle data recorders (VDRs) on a subset of emergency service vehicles (5–12 vehicles from each department) to assess the utility of collecting driving data and explore ways to proactively use such data to prevent crashes. Vehicle data recorders (VDRs), similar to ‘blackbox’ systems found in commercial airliners, have recently been required as standard equipment on all new fire vehicle apparatuses per the NFPA1901 standards. VDRs collect second-by-second engine data (e.g., vehicle speed, acceleration, anti-lock braking system events, time, date, etc.) in a continuous 48-h loop. However, to access and store these data for review, special software and a hardwired connection is required to download the data every 48 h of engine time, making data collection time consuming and cumbersome. To streamline data collection, we decided to use a cellular telematics system to wirelessly collect and store VDR data in cloud accessible servers.

Telematics tracking is a system that collects real time driving data from the onboard vehicle computer and transmits it wirelessly via cellular networks to an online server for review. The telematics system we used permitted the collection of standard VDR data in addition to other data metrics not commonly available on stock VDR systems such as, driver and passenger seatbelt status, vehicle location (via GPS), and a set of lateral and longitudinal g-force measures to assess hard acceleration, hard cornering and harsh braking patterns. Email alerts were sent to safety managers when driving rules were exceeded (e.g., driving over speed limit). The telematics data were intended to be used to monitor driving behaviors and identify areas for driver training. Telematics tracking for this study was accomplished using a commercial vendor.

### Process evaluation

The RM process was well received and all departments completed the process within the planned schedule of meetings. Dedicated time to systematically focus on improving safe driving and brainstorming interventions to reduce ESVCs was a universally cited benefit (Table 3). The RM teams cited the review of departmental crash data during the scoping process to be useful in understanding crashes. Department C cited the RM process as beneficial in organizing their own safety review practices into a more formal and defined workflow.

**Table 3:** Selected Controls and Summary of Perceived Risk Management Implementation Benefits and Challenges by Department

Dept.	Selected Controls	Benefits	Challenges
A	• Side and rear view cameras on ambulances* • Modified Code 3 dispatch SOP and policies* • New Backing SOP • Telematics monitoring • Train the trainer driving course* • Increased driving simulator use • Increased remedial driver training frequency* • Daily safety simulcast messages* • Removed garage door clickers from stations*	• RM allowed for proactive and focused review of crashes, safe driving and associated risks and hazards • Facilitated adoption of controls and change in policies based on data	• Disagreements between union and administration on adopting potentially punitive control measures (monitoring driver telematics data).
B	• Back up cameras and proximity sensors • In-cab headsets for driver-to-spotter communication • Modified Code 3 dispatch SOP and policies • Telematics monitoring* • Increased simulator use • Targeted quarterly training based on crash data priorities* • Mapping locations of prior deer collisions to identify hotspots*	• Enjoyed RM process and discovery of risks and hazards for crashes • Found RM useful for brainstorming and identifying needed controls not considered previously	• Found it difficult to evaluate large amounts of driving/crash data • Lack of county support to purchase new interventions and safety equipment • Limited involvement of volunteer firefighters
C	• Telematics monitoring* • Back up cameras and proximity sensors* • Modified Code 3 dispatch SOP and policies*	• RM was useful for reviewing current controls and gaps • Enhanced conversation about driving behavior and crashes • Provided formalized framework for managing risks	• Difficult to show changes/effectiveness because already had few crashes • Limited resources and personnel to review data

*asterisks indicate installed or implemented

Although the RM process was perceived to be generally useful by all three departments, there were acknowledged challenges during the implementation phase. In one department there was strong union concern about potential use of telematics data for punitive disciplinary actions on drivers. Another department reported bureaucratic barriers to implementation of controls, such as difficulty in getting approval from the district or budget constraints. In the suburban department, volunteer firefighter involvement was low and it was not possible to include volunteer firefighters throughout the RM meetings. The rural fire department noted it was difficult to evaluate the effectiveness of the program since no ESVCs had occurred in over 7 years prior to the study (the incident involved an unmarked staff vehicle). Finally, across all departments, the large amount of detailed telematics data provided in stock formats (i.e., through email alerts and excel spreadsheets) by the telematics company was difficult to review and not immediately useful.

## Discussion

RM has been used in the fire service to reduce occupational injury and associated economic costs [14, 15]. Previous research shows that RM is well-accepted by firefighters and that specific components of the RM process, especially the participatory approach and analysis of risks associated with specific tasks, are useful to and valued by firefighters [14]. In this study, we applied formalized RM to address ESVCs in our partner fire departments, which was a new application of the approach.

Emergency response driving was cited as a major concern across all departments and is a frequently cited risk factor in prior studies. Donoghue et al. reported that 66% of firetruck crashes occurred during emergency response [5]. Custalow and Gravitz reported that 91% of emergency vehicle collisions in Denver occurred under lights and sirens [17]. While the exact mechanisms that increase a driver’s risk of collision during emergency response is not completely clear, the association does seems to be driven by generally higher speeds and reduced reaction times during emergency responses [16]. Savolainen et al. reported emergency response crashes to be common in Michigan and cited speeding, overtaking, and passing to be common factors contributing to the risk of these collisions [18]. High speeds reduce driver’s ability to react to hazards, safely control the vehicle, and navigate traffic. Policy changes reclassifying certain categories of calls as not requiring an emergency response have reportedly proven effective in reducing ESVCs in other fire departments like in St. Louis, Virginia Beach and Phoenix [16] .

Poor visibility due to weather, road environment, and apparatus design were medium to high priorities for all fire departments. Visibility concerns were often raised in conjunction with backing incidents since driver’s rearward field of vision is practically obscured in all fire apparatuses. Fortunately, back up cameras and rear facing cameras have been found to be generally effective in reducing backing related incidents in civilian drivers, though their use has been rarely if ever tested in the emergency services [19, 20]. Clarke et al. found that emergency responders in the UK had generally low “blame worthiness” levels in emergency response collisions; however, failure to check blind spots was the most common factor in at-fault collisions [21]. Furthermore, Custalow and Gravitz have noted that visual clearance of intersections is necessary in reducing intersection collisions which have historically been the crash type associated with the highest risk of injury [17]. Department C reported that driver-vehicle incompatibility to be common concern since a driver’s field of vision can be diminished in fire apparatuses. This problem of driver-vehicle incompatibility is particularly exacerbated when drivers are not assigned to permanent vehicles which they may customize to fit their driving style [16].

Insufficient driver training and education was also cited as a common concern across all partner fire departments. Driver error has been cited as a primary cause in up to 93% of ambulance-involved collisions; thus, training remains a first-line priority for all departments despite being inconsistently applied and minimally required [22]. While NFPA 1002: *Standard for Fire Apparatus Driver/Operator Professional Qualifications* provides minimum job performance standards for emergency vehicle operators, the standards are not legally required nor enforceable. The only legal requirement is that drivers are trained to meet minimum state and federal laws for operating large heavy trucks. Moreover, since 1986, virtually all states have exempted emergency responders from Commercial Driver’s License (CDL) requirements which was designed to discourage unsafe drivers from operating large commercial vehicles [16]. Training space is also another concern, as Department A reported the lack of dedicated training spaces as a critical limitation to their hands-on driver training program. Dedicated driving space is an important feature in driver training, since training conducted on closed ranges have been associated with a reduction in crash risk over traditional on-road driver training [23].

A key advantage of RM—in contrast to compliance based safety programs—is the emphasis on establishing context and tailoring interventions to site-specific needs. The advantage of considering context and needs in selecting controls is that limited resources may be concentrated towards targeted interventions to address high priority risks instead of broad target interventions that may have only secondary or tertiary effects on a specific high priority need. The needs between urban and rural fire departments are particularly prescient given they operate in very different conditions [22]. This important advantage of RM was evident in the notable differences in prioritized risks and incidents in our dense urban departments and rural fire departments. The fire departments with more urban environments tended to prioritize risks and hazards associated with driving in traffic congested areas, while rural departments were more concerned with animal collisions and environmental hazards such as inclement weather on less developed roads. This is consistent with prior work by Ray and Kupas where they found that compared to urban settings, rural ambulance crashes tend to be more likely on snowy roads (13% vs 5%) and at night without street lighting (25% vs. 4%) [22]. Moreover, in comparison to rural departments, urban departments tended to experience more angled collisions with other vehicles (54% vs 19%), intersection collisions (57% vs 36%) and incidents at traffic signals or stop signs (53% vs. 14%), which is consistent with the risks experienced by drivers in dense urban areas [22]. Nearly all volunteer firefighters (95%) work in suburban and rural departments serving populations under 25,000, facing different training and working conditions than urban-based career firefighters [24]. RM scoping and risk assessments should account for volunteer firefighters responding from home, and factor in differences in apparatus training programs and driving experience.

A potential limitation of the risk assessment process that we used was the reliance on the perceived likelihood and severity of incidents to rank and prioritize vehicle incident types. While experiences and perceptions are valid, data driven assessment may provide more objective and substantive rankings of hazards to be controlled. However, given that data are often not available or poorly collected, opinion-based risk assessments are generally the norm, particularly in cases where the involved team members clearly understand the situations, operations and risks being reviewed [9, 14]. Risk matrices are rarely validated in their ability to improve risk management decisions, and are prone to poor resolution (i.e., very different risks can have the same ranking) as well as errors where a qualitatively ranked risk may be high with very low quantitative risk [25]. Another key limitation to our study is that the departments self-selected to participate in the RM process, resulting in a potential for selection bias. Though the effects of selection bias may be difficult to quantify, it is expected that the personnel that elected to participate in our study may differ from the general emergency service population (for instance in education or experience), thus the identified priorities and hazards during the risk assessment may not be representative of true priorities and hazards in the emergency services (i.e., external validity is limited). Regarding Department B, the crash data reviewed during scoping were limited to only career firefighters, thus inferences about risks and hazards in this combination department does not reflect the volunteer firefighter force.

Education and training featured prominently as a control adopted by our partner fire departments. Given the paramilitary nature of firefighting organizations and skill-based operations, it was not surprising that drivers training was a commonly advocated strategy to improve driver safety [16, 17]. Although the evidence base is mixed, prior studies suggest that licensing programs for young novice drivers may reduce the risk of fatal crash involvement, particularly in graduated driver licensing programs where the learning period and amount of practice is extended [26–28]. The effectiveness of graduated licensing programs in civilian populations suggests graduated driver training programs where independent driving responsibilities are gradually increased may be effective in reducing the risk of crash involvement in the fire service.

It should be noted that published evaluations of driver training programs have predominantly been conducted on young novice drivers with little to no driving experience—not emergency services populations. We have found no published studies or reports evaluating the effect of emergency service vehicle operations training and recommend these programs be evaluated. In our collective experience working with the fire service, we have found initial fire service driver training programs to be variable in duration, availability, quality and curriculum, rendering the evaluation of fire service training programs difficult.

Though initial drivers training and licensure is important in developing safe driving skills, there have been no published studies to indicate post-licensure training or remedial training to be effective in reducing crashes in drivers that have already been trained [29, 30]. In a recent systematic review, Masten and Peck report that driver remediation is only slightly effective in reducing road crashes among civilian drivers, but most of the benefits came from driver suspension programs and only then could be due to more careful driving in the post suspension intervals evaluated [30]. In a recent meta-analysis of 21 randomized controlled trials studying the effects of post-licensure driver education, Ker et al. found small non-significant reductions in road traffic crashes among drivers receiving post-licensure training [31]. Custalow and Gravitz have found 71% of drivers involved in emergency response collisions had a history of multiple collisions, suggesting that remedial training may not be effective since drivers sent to remedial training remain at risk for repeated collisions [17]. These findings suggest that reliance on remedial training should be tempered and used in tandem with other approaches to reduce the risk of collision. Furthermore, there is evidence to suggest that training programs which focus on changing risk perceptions and reducing risk tolerance in additional to driving competency may be more effective than traditional skill-based training programs in reducing the future risk of crash involvement [28].

The bulk of challenges in the RM process occurred during the implementation of controls. The potential for using telematics driving data in a punitive fashion was a great concern for department union leaders. Engagement of union representatives and members from the outset is critical to a successful RM program, especially one targeting driving and crashes where fault and liability may prompt punitive action as opposed to education and proactive prevention. Simulator training in Departments A and B was hampered by reports of trainee nausea caused by ‘simulator sickness,’ which is a commonly reported negative side effect of stimulator use [32]. As previously stated, the format of the telematics driving data was provided directly from the company of the fire department partners. The data provided were too detailed for the safety managers and required substantial time to review and analyze. Based on feedback from our fire department partners, we are developing weekly visual ‘dashboard’ summary reports for each department which provides fleet-level as well as vehicle-level statistics of driving performance. The dashboard reports summarize miles driven, trips driven, driving rule exceptions, and ranks each vehicle against each other based on the number of driving rule exceptions per mile. The report is emailed to the safety department on a weekly basis. Each dashboard is customized to meet the needs of the department and what they deem important to safety and operations. We strongly recommend that departments planning to incorporate VDR or telematics data into a comprehensive safety program engage a vendor that can customize data reports and visualizations based on specific department goals and needs.

## Conclusions

Risks and hazards for ESVCs are site dependent. Proactive RM introduced in our three partner fire departments was considered, by the RM teams, a beneficial and acceptable process, allowing each fire department to implement controls tailored to their own priority hazards. The findings of this study support the broader use of RM to increase the knowledge and understanding of ESVCs in the US fire service.

## Additional file

Additional file 1: Risk Management Process and Impact Evaluation Questions. The semi-structured interview guide used during the risk management process evaluation. (PDF 201 kb)

## Abbreviations

CDL: Commercial driver license
ESVC: Emergency service vehicle crash
NFPA: National fire protection association
RM: Risk management
US: United States
VDR: Vehicle data recorder
