# Correction to: Risk management of emergency service vehicle crashes in the United States fire service: process, outputs, and recommendations

**DOI:** 10.1186/s12889-017-4946-8

**Published:** 2017-12-01

**Authors:** David P. Bui, Keshia Pollack Porter, Stephanie Griffin, Dustin D. French, Alesia M. Jung, Stephen Crothers, Jefferey L. Burgess

**Affiliations:** 1 Mel and Enid Zuckerman College of Public Health, The University of Arizona, Drachman Hall, 1295 N Martin Ave, Campus PO Box: 245210, Tucson, AZ 85724 USA; 2 Johns Hopkins Bloomberg School of Public Health, Johns Hopkins Center for Injury Research and Policy, Baltimore, MD USA; 3 Center for Healthcare Studies, Department of Ophthalmology, Northwestern University Feinberg School of Medicine, Chicago, IL USA; 4 Department of Veterans Affairs, Center of Innovation for Complex Chronic Healthcare, Edward Hines, Jr. VA Hospital, Hines, IL USA; 5 Seattle Fire Department, Seattle, WA USA

## Correction

After publication of the article [1], it has been brought to our attention that the second author’s name was published incorrectly. Previously included as “Keshia P. Porter”, the full and correct name should be “Keshia Pollack Porter”. This has now been corrected in the original version of the article.
