# Supplementary material for: Risk management of emergency service vehicle crashes in the United States fire service: process, outputs, and recommendations
Source: BMC Public Health. 2017 Nov 17;17:885. doi: 10.1186/s12889-017-4894-3 (PMC5693554; doi:10.1186/s12889-017-4894-3)
Supplement: Additional file 1: — Risk Management Process and Impact Evaluation Questions. The semi-structured interview guide used during the risk management process evaluation. (PDF 201 kb) [file 12889_2017_4894_MOESM1_ESM.pdf]

Risk Management Process and Impact Evaluation Questions

Introduction

Hello. My name is [*interviewer*] and I am [*include affiliation*]. I am working with the University of Arizona Team on this research.

The purpose of our discussion today is to hear from you how the risk management process was implemented; how its working, challenges that were encountered, and lessons learned. Our discussion is very important since it will generate information to guide future replication of this intervention strategy.

As a reminder, your participation is voluntary. And since you are volunteering to be here, I ask that you are candid and that you view this as a discussion. I will not record your names, and things that you say will not be tied to your name in the products that we create based on this work. I will summarize what you say with the other groups and individuals that I am speaking with, and report the common themes. Similar to the other sessions, with your permission, I will be recording this session; thus, please remember to speak loud and clear, and one person at a time.

Does anyone have any questions? Then let's begin.

Let's talk about your thoughts on risk management process thus far:

What are your thoughts about the risk management process that was used to develop the interventions that were implemented?

Probe, as needed:

Was it a useful approach for preventing injuries?
What were some of the challenges with this approach?
What were some of the strengths of this approach?
Is there another approach to improve injury that you would have rather tried instead?

After participating in the risk management process, do you think differently about firefighting risks?

Probe, as needed:

How so?
Ask for an example

Have you changed your behavior/activities based on this process?"

Implementation *<u>(these questions will not be relevant for everyone)</u>*

Tell me about how the strategies were implemented

Probe, as needed:
What kinds of challenges have arisen during the implementation process?
How were those challenges addressed, if they have been?
What administrative processes are in place to support implementation?
Who are the main stakeholders involved in the implementation process?

What is working well?
What is not working well?

How can the implementation process be improved?
What resources are being devoted to oversight of the control strategies?
Do you have a sense for how much implementation is costing?

What do you predict will happen to the interventions once the research study is complete?

What have you learned from the implementation of this policy that would be helpful to others who are interested in preventing injuries using the risk management approach?

Reinforcing Behavior

What could the fire department do as an organization to reinforce the safety behaviors learned through the risk management process?

Do you see a role for incentives to motivate departmental safety behavior?
If yes, what are some possible motivators that might reinforce behavior?
If no, why not?

What about peer leaders?
Why or why not?

Now that you have completed the risk management process, I want to hear your thoughts on injury prevention. *(These are for the original participants)*

Do you believe that all injuries during firefighting are preventable?

Do you believe that getting injured during firefighting activities is simply “part of the job”?
Do you believe that you have control over your own risk of sustaining an injury while working as a firefighter?

Do you feel that the risk management approach was a good way to identify all of the risks? If so, then why? If not, then why not?

Do you think that control strategies that were implemented through the risk management process created a safer work environment for all of those who are part of the fire department?

Probe, as needed:

Why? Or Why not?

If yes, then try to get to the how, how did it create a safer environment.

Replication and Sustainability

If we were going to try this same process with another fire department, in your opinion, what do they need to know about this process?

If the fire department were to adopt this "bottom up," risk management process, what other operations/job tasks do you feel are appropriate for developing injury control strategies (e.g., drilling/training, technical rescues, etc)?

Do you believe the involvement of university/outside experts in setting up and evaluating the risk management process was helpful or necessary?

Do you feel the process can now continue without them?

Conclusions

Is there anything else that you want to tell me about this process?

Thank you for speaking with me.
